# A Case of Entamoeba histolytica Liver Abscess in an Immigrant Patient

**DOI:** 10.7759/cureus.53902

**Published:** 2024-02-09

**Authors:** Nivedha Balaji, Aleksandra Ignatowicz, Sheena Bhushan, Vaishali Jadhav

**Affiliations:** 1 Internal Medicine, Northeast Georgia Medical Center Gainesville, Gainesville, USA

**Keywords:** entamoeba histolytica infection, immigrant, liver abscess, amebic liver abscess, entamoeba histolytica

## Abstract

*Entamoeba histolytica* is a pathogenic protozoan endemic to Asia, Africa, and Central and South America. However, with increased travel and emigration, it is becoming a common parasitic infection leading to many worldwide deaths. We present a case of a young Hispanic male immigrant with an amebic liver abscess. This case report highlights the complexities of diagnosing and treating *E. histolytica* infection.

## Introduction

*Entamoeba histolytica* is a protozoan responsible for amebic dysentery, colitis, and liver abscess [1-3]. Amebiasis is endemic to countries in Asia, Africa, and Central and South America. Intestinal and hepatic amebiasis is endemic in Venezuela [4]. Presenting symptoms are vague and include abdominal pain, diarrhea, weight loss, and fever. Since amebic liver abscess is quite rare in North America, diagnosing it can be quite challenging. Prompt and appropriate treatment is necessary to prevent life-threatening complications, including necrotizing colitis, toxic megacolon, perianal ulceration, colonic perforation, and even hematogenous spread to the brain and lungs [2,5,6]. We describe a case of amebic liver abscess that presented with abdominal pain without diarrhea.

## Case presentation

A 34-year-old male presented to the emergency department with generalized abdominal pain, fevers, loss of appetite, night sweats, and 10 lbs. weight loss over one-month duration. He denied any diarrhea, prior diarrheal illness, any notable ova or parasites in his feces, unusual food consumption, occupational exposures, and family history of any cancers. Social history was remarkable for recent emigration from Venezuela to the United States three months ago. He denied having any sick contacts. On arrival, his vital signs were a heart rate of 110 beats/minute, respiratory rate of 20 respirations/minute, saturating at 98% on room air, blood pressure of 114/67 mmHg, and temperature of 36.9°C. Physical examination was remarkable for mild tenderness in the right upper quadrant (RUQ). Significant laboratory values are listed in Table 1.

**Table 1:** Laboratory values

Component	Patient’s value	Normal range
Aspartate aminotransferase	57 U/L	0-48 U/L
Alanine transaminase	131 U/L	13-61 U/L
Alkaline phosphatase	632 U/L	45-136 U/L
Total bilirubin	0.3 mg/dL	0-1 mg/dL
Erythrocyte sedimentation rate	107 mm	0-15 mm
C-reactive protein	9.70 mg/dL	0-0.6 mg/dL
Hemoglobin	9.9 g/dL	14-18 g/dL

**Figure 1:**
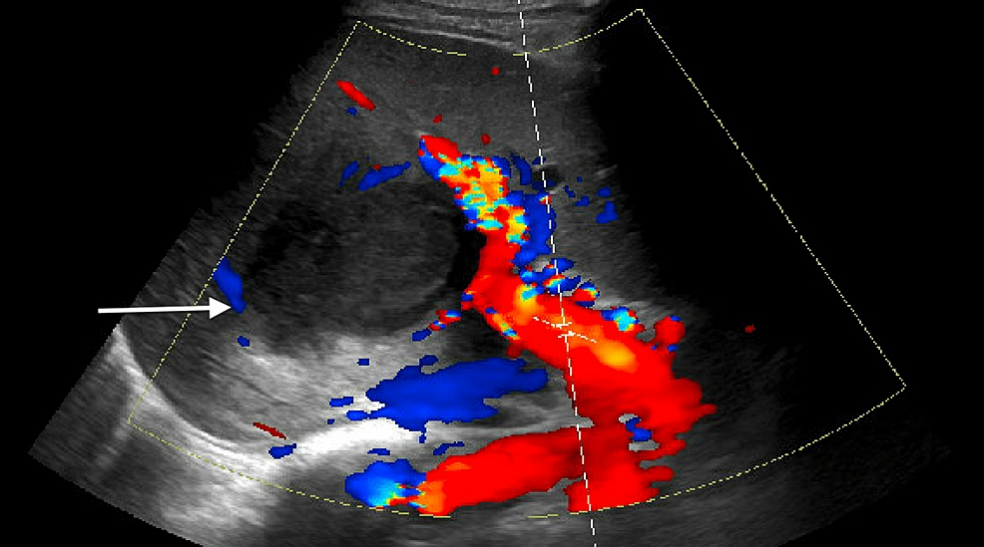
Right upper quadrant ultrasound Right upper quadrant ultrasound showing a mildly vascular lobulated hypoechoic mass in the right hepatic lobe measuring 11.7 x 9.7 cm.

Blood cultures, stool ova and parasites, and HIV testing were unremarkable. RUQ ultrasound showed a right hepatic lobe mass (Figure 1).

**Figure 2:**
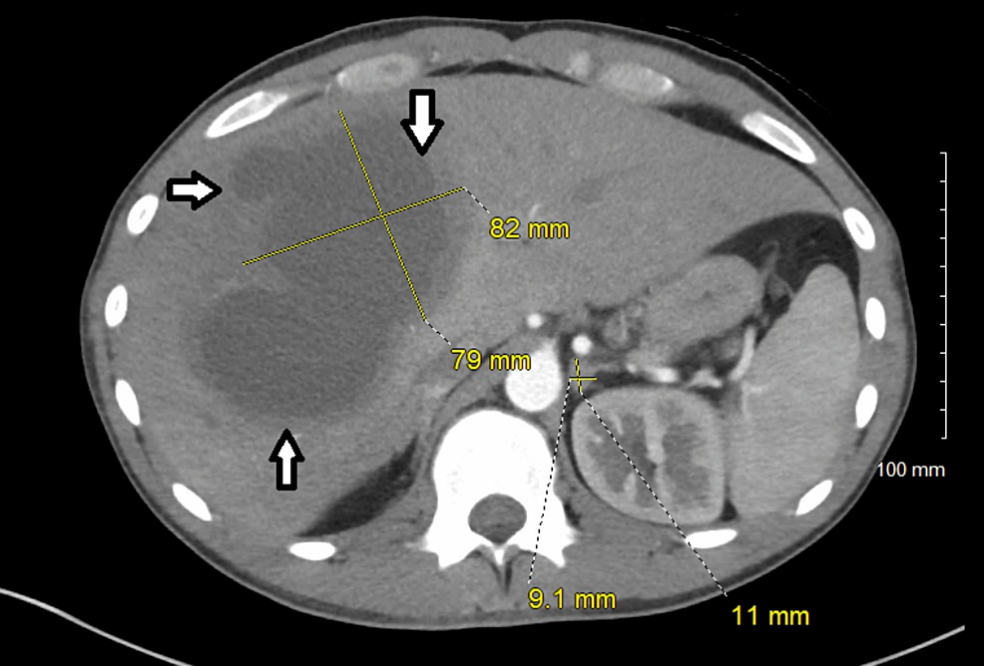
CT of the abdomen and pelvis CT of the abdomen and pelvis with contrast showing the anterior-posterior view of multiple hypoenhancing hepatic lesions measuring 79 x 82 mm, 78 x 55 mm, and 24 x 30 mm, and a left adrenal nodule measuring 11 x 9.1 mm.

**Figure 3:**
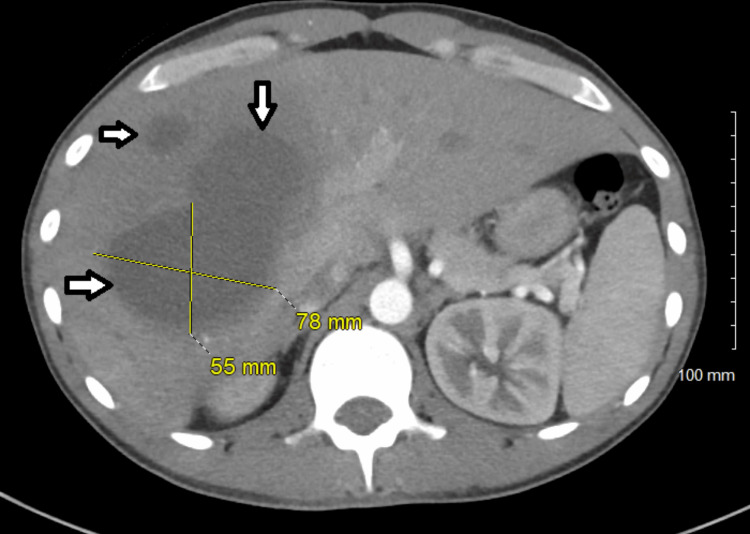
CT of the abdomen and pelvis CT of the abdomen and pelvis with contrast showing the anterior-posterior view of multiple hypoenhancing hepatic lesions measuring 79 x 82 mm, 78 x 55 mm, and 24 x 30 mm, and a left adrenal nodule measuring 11 x 9.1 mm.

Computed tomography (CT) of the abdomen and pelvis with contrast showed three large hypodense hepatic lesions with central fluid density and a left adrenal nodule (Figures 2, 3).

Surgery was first consulted while the patient was in the emergency department. The surgery team recommended against aspiration of the hepatic lesions due to an increased risk of perforation and contamination and due to the patient being hemodynamically stable. They mentioned that the patient could avoid the risk of perforation and be treated with outpatient antibiotics and antiparasitic therapy. The infectious diseases team was consulted for medical management. They empirically started him on albendazole 400 mg twice daily and piperacillin-tazobactam 4.5 g every six hours. Meanwhile, the patient’s serology returned positive ELISA (enzyme-linked immunosorbent assay) for *E. histolytica* antigen and was negative for *Echinococcus* antigen. The patient’s antibiotics were switched to metronidazole for 10 days followed by paromomycin for seven days to ensure parasitic eradication from the GI tract. Approximately one month post-ED arrival, his symptoms had resolved. A repeat *E. histolytica* antigen was negative. However, a repeat RUQ ultrasound showed a persistent complex fluid collection in the right hepatic lobe warranting follow-up. Unfortunately, the patient failed to follow up with infectious diseases and surgery after this visit.

## Discussion

*E. histolytica* is typically transmitted by the fecal-oral route and sexual intercourse. Upon contamination, there is excystation of the parasite within the small intestines, followed by the invasion of the trophozoites that lyse the colonic epithelium. As part of the inflammatory process, neutrophils then further damage the colonic epithelium leading to extraintestinal contamination via hematogenous spread to the liver and peritoneum resulting in further necrosis, abscess formation, and perforation [2,7-9].

Amebic colitis presents with abdominal pain and watery or bloody diarrhea that can range from mild to severe dysentery. Amebic colitis symptoms are nonspecific and can present similar to those of infections from *Shigella*, *Salmonella*, *Campylobacter*, *Escherichia coli*, diverticulitis, inflammatory bowel disease, intestinal tuberculosis, and ischemic colitis. Amebic liver abscess presents with fever, bloody diarrhea, and signs of peritoneal irritation. Hematogenous or lymphatic spread from intestinal lesions to the pericardium can present with chest pain, shortness of breath, and edema. Extension to the lungs can result in pulmonary amebiasis causing fever, hemoptysis, and referred pain to the right shoulder or intrascapular region as the right lower or middle lobe is the most common region of spread [5].

Diagnosis can be delayed owing to a lengthy latency period between infection and clinical symptom onset. Evaluation includes fecal microscopy, fecal and/or stool antigen, fecal polymerase chain reaction (PCR), and histopathologic examination of colonic biopsy. Fecal microscopy is the first line of investigation but offers suboptimal sensitivity to identify cysts and trophozoites with sensitivity <60% [2,3]. It should be avoided if other diagnostic means are available. Stool PCR is the gold standard and has >70% sensitivity and >90% specificity. Stool antigen testing has 90% sensitivity, but serum antigen testing has 65% sensitivity. Detection of antibodies is possible in approximately 70-90% of individuals within five to seven days of infection [2]. Direct visualization via colonoscopy can help identify “flash-like” ulcerations or erosions, bloody exudates, edematous mucosa, cryptitis, cryptitis, and crypt abscesses [5]. Serologic testing remains the gold standard for hepatic amoebiasis. Additional ultrasound and CT imaging of the abdomen, pelvis, brain, and chest may be used to assess invasive and extra-intestinal infections [9].

While approximately 90% of cases are self-limiting and asymptomatic, the complications associated with *E. histolytica* include amebic liver abscess, fulminant necrotizing colitis, toxic megacolon, and fistulizing perianal ulcerations when timely diagnosis and management is not conducted [2,5]. The mortality rate increases to 40% with the development of necrotizing colitis and 89% with a concomitant liver abscess. Concomitant corticosteroid use has been linked to the development of toxic megacolon, which requires emergency surgical intervention [5].

Noninvasive colitis should be treated with a luminal agent like paromomycin to eliminate intraluminal cysts but invasive amebiasis and extraintestinal disease should be treated with nitroimidazoles. However, 40-60% of patients can present with persistent intestinal parasites after nitroimidazole; hence, treatment with nitroimidazole should be followed by paromomycin to prevent relapse. Second-line agents like diiodohydroxyquin and diloxanide furoate are available alternatives. Broad-spectrum antibiotics can be added to the treatment for fulminant amebic colitis. Surgical intervention is necessary for patients who develop acute abdomen or toxic megacolon. Therapeutic aspiration or catheter drainage can be used for abscesses with a high risk for rupture larger than or equal to 5 cm or in the left lobe or pleural effusions [5]. The patient presented in this case report had hepatic lesions that were present in the right lobe, and he was hemodynamically stable. The surgery team recommended against aspiration of the hepatic lesions as there was no emergent need to have the patient undergo a procedure that could increase the risk of lesion rupture and contamination. Finally, clinicians should educate patients to minimize fecal-oral transmission and prevention of amebiasis by encouraging personal hygiene practices [5].

## Conclusions

Due to the low overall prevalence in the Western world and the long latency period between infection and symptom onset, *E. histolytica* diagnosis can be delayed leading to inadequate management. Owing to globalization, *E. histolytica* must be considered as a potential cause of liver abscess, especially in immigrants and patients with a travel history to endemic countries. A thorough social and travel history is paramount for early diagnosis and management. It is also essential to recognize that *E. histolytica* can masquerade as other common medical conditions with vague clinical symptoms.
